# P02.33. Effects of inpatient integrative medicine on pain and anxiety in an acute cardiovascular population

**DOI:** 10.1186/1472-6882-12-S1-P89

**Published:** 2012-06-12

**Authors:** A Fyfe-Johnson, P Johnson, N Ghildayal, J Dusek

**Affiliations:** 1 Penny George Inst. for Health & Healing, Abbott Northwestern Hospital, Allina Health, Minneapolis, USA; 2 Center for Healthcare Innovation, Allina Hospitals & Clinics, Minneapolis, USA

## Purpose

To explore the impact of integrative medicine (IM) on pain and anxiety in an acute cardiovascular inpatient population.

## Methods

Primary observational data were collected on 280 inpatient admissions that: (1) had a primary diagnosis of ischemic heart disease, acute cardiovascular (CV) disease, or other forms of heart disease, and (2) were seen by IM staff at Abbott Northwestern Hospital. IM providers collected data before and after each treatment session; primary outcomes were pre and post-treatment pain and anxiety scores using a 0-10 numeric self-report intensity scale. Data were retrospectively analyzed from the IM database after electronic health record extraction for calendar year 2010. Chi-square tests were used to test for significant differences in the percentage of patients with acute cardiovascular conditions who received specific IM therapies, while paired t-tests were used to test for significant differences in mean pain and anxiety score changes in the same population.

## Results

Pain management in acute care hospitals is a major institutional therapeutic challenge; the stress-pain relationship is particularly relevant to the acute CV population. Overall, there was a marked decrease in pain (47.9%, p<0.001) and anxiety (42.2%, p<0.001) scores for acute CV patients receiving IM therapies (n=280). Massage was most utilized (55.7%), followed by aromatherapy (40.4%), and mind/body therapies (39.6%) for acute CV conditions (n=280). Reductions in pain and anxiety were all significant (p<0.001) after stratifying by most common acute cardiovascular diagnoses: ischemic heart disease, acute CV disease, or other forms of heart disease. The greatest reductions in pain (52.9%, p<0.001) and anxiety (48.4%, p=0.001) were observed in patients with ischemic heart disease (n=87).

## Conclusion

IM appears to be effective for reducing pain and anxiety in acute inpatient cardiovascular conditions. These findings provide incentive to further investigate mechanism(s) of pain and anxiety changes in an acute cardiovascular population.

